# Ruptured desmoid tumor imitating acute appendicitis – a rare reason for an emergency surgery

**DOI:** 10.1186/s12893-019-0662-x

**Published:** 2019-12-16

**Authors:** Yavor Asenov, Stoyan Genadiev, Alexander Timev, Jeni Panaiotova, Valeria Hadjiiska, Tihtchev Veselin, Theophil Sedloev

**Affiliations:** 1 Department of Surgery, Medical University – Sofia, University Hospital “Queen Joanna”, 8 “Byalo More” Str., 1527 Sofia, Bulgaria; 2 Department of Vascular Surgery, Multi-profile Hospital for Active Treatment “Uni Hospital”, 100 “Georgi Benkovski” Str., 4500 Panagiurishte, Bulgaria; 3 Department of Urology, Medical University – Sofia, University Hospital “Alexandrovska”, 1 Georgi Sofiiski blvd, 1431 Sofia, Bulgaria; 4 Department of Gynecology, Multi-profile Hospital for Active Treatment “Nadezhda”, 3 “Blaga vest” Str., 1330 Sofia, Bulgaria; 5 Department of Nuclear Medicine, Medical University – Sofia, University Hospital “Alexandrovska”, 1 “Georgi Sofiiski” blvd, 1431 Sofia, Bulgaria; 6 Department of Pathology, Medical University – Sofia, University Hospital “Queen Joanna”, 8 “Byalo More” Str., 1527 Sofia, Bulgaria

**Keywords:** Desmoid tumor, Aggressive fibromatosis, Fibromatosis, Intestinal mesentery, Soft-tissue sarcomas, Case report

## Abstract

**Background:**

Desmoid tumors, also known as aggressive fibromatosis, are extremely rare, accounting for less than 3% of soft-tissue sarcomas and less than 0,03% of all neoplasms. The diagnosis is usually delayed because of the lack of specific symptoms, and can sometimes lead to serious and, even fatal complications.

**Case presentation:**

We report the case of a 27-year-old male patient presenting with the clinical picture of acute appendicitis. During the operation, we found a tumor in the jejunum with a necrotic zone and perforation on its surface, causing hemorrhagic effusion into the abdominal cavity and subsequent peritonitis. The tumor was removed with negative margins via resection of the small bowel. The final histological result showed aggressive fibromatosis.

**Conclusions:**

Aggressive fibromatosis remains a serious problem with the possibility of locally aggressive behavior with high rates of recurrence. Sometimes, its clinical and macroscopic recognition can be immensely tricky. As shown by our patient, on rare occasions, desmoid tumors can lead to acute surgical abdomen requiring an emergency operation.

## Background

Desmoid tumors, also known as aggressive fibromatosis (AF), are extremely rare pathologies, accounting for less than 3% of soft-tissue sarcomas and less than 0,03% of all neoplasms [1]. They can occur sporadically or as a part of congenital syndromes (Gardner’s syndrome, familial adenomatous polyposis - FAP, and bilateral ovarian fibromatosis) [2]. Desmoid tumors originate from musculoaponeurotic structures and have dual behavior. Although these tumors are benign neoplasms without metastatic potential, they can affect every part of the body, can be locally aggressive and have a high recurrence rate [3, 4].

Desmoid tumors remain a diagnostic and therapeutic problem and a highly individualized treatment approach by expert teams is required. Due to the rarity of the disease, the level of evidence available for common types of cancer is unlikely ever to be available for it [5]. Even more challenging are situations in which the aggressive fibromatosis leads to peritonitis, requiring emergency operations. We present a patient with a perforated intraabdominal desmoid tumor with hemoperitoneum and peritonitis mimicking acute appendicitis. To the best of our knowledge, this is the first such case reported in the literature.

## Case presentation

The case report was prepared following CARE guidelines. We present a 27-year-old male patient with complaints of pain in the lower right abdominal quadrant and suprapubic area with a duration of 4–5 h. The pain radiated to the right scrotum, and the patient noticed mucus at the end of micturition. Initially, the pain was colic, but at the moment of the physical examination, it was permanent, without nausea or vomiting. The patient reported an episode of fever up to 37,5 °C 2 days before, which quickly passed. The patient had no comorbidities or previous surgical procedures. The laboratory tests showed leukocytosis – a white blood cell count of 14,6 G/L, mild anemia – a hemoglobin level of 101 g/L, a red blood cell count of 3,5 T/L, a hematocrit level of 0,32; other parameters were within normal ranges. A urine test revealed the presence of protein, and there were red and white blood cells in the sediment. The X-ray of the abdomen showed only one air-fluid level with a small bowel origin. Ultrasound imaging did not demonstrate liquid behind the urinary bladder or additional abdominal pathology. Based on the findings, a diagnosis of appendicitis was suspected with the differential diagnosis of urinary tract disorders with cystitis. We admitted the patient to the hospital and began treatment with infusions of saline solutions, spasmolytics, and antibiotics. Despite this, the abdominal pain increased during the next 4 h, and signs of positive rebound tenderness (Blumberg’s sign) appeared.

**Fig. 1:**
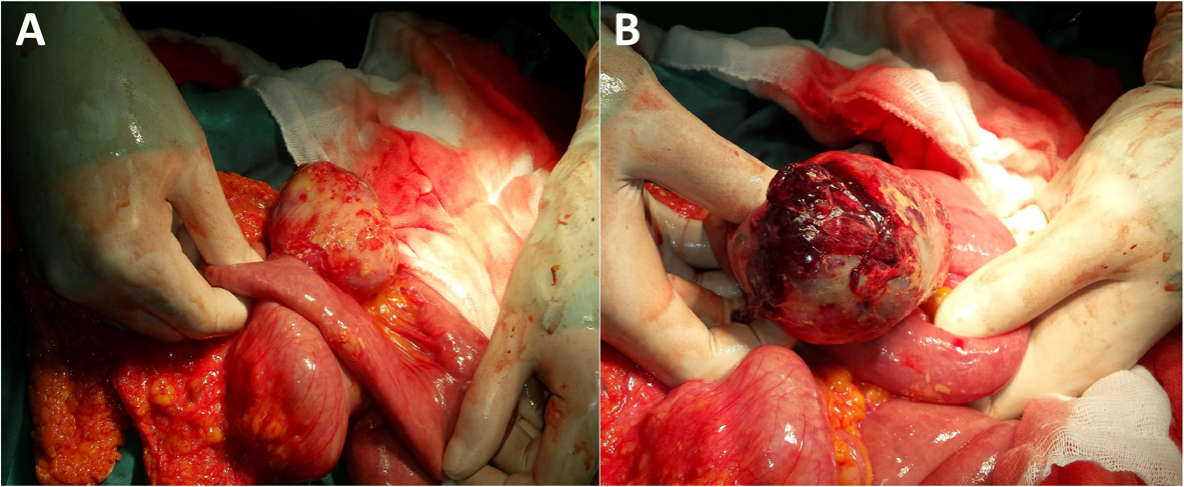
**a** Intraoperative findings; **b** Focus on the necrotic zone in the tumor

**Fig. 2:**
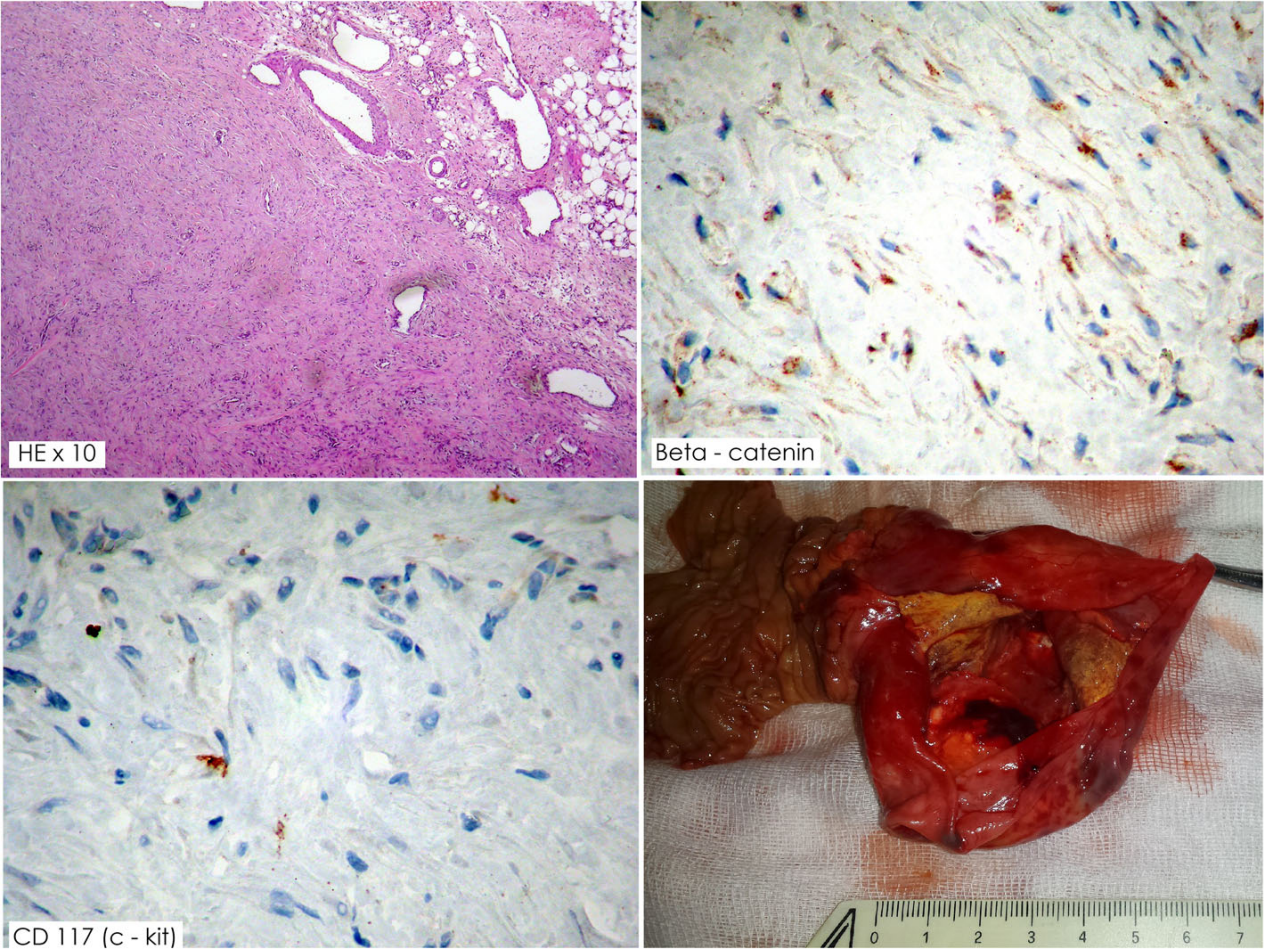
Histological and Macroscopic view of the specimen; Immunohistochemical tests positive for β-catenin and negative for CD117 (c- kit)

Therefore, we decided to proceed with surgery without any further imaging investigations due to the highly probable diagnosis of acute appendicitis with spreading peritonitis. Abdominal exploration revealed a serohemorrhagic effusion of approximately 550 ml, which was aspirated. Surprisingly, a tumor formation involving the jejunum in its proximal third was found. The affected loop was situated near the ileocecal confluence. The mass consisted of cystic and solid areas. Macroscopically, it was difficult to determine the tumor origin – from the mesentery or the intestinal wall. In the cystic part, there was a necrotic zone with perforation, explaining the presence of hemorrhagic effusion in the abdominal cavity (Fig. 1a, b). The tumor was removed via resection of the small bowel, and the ex vivo dissection revealed a solid mass with ulceration located in the cystic sack (Fig. 2).

The histopathology of the specimen revealed an aggressive fibromatosis, which was removed with negative margins. The patient had no history of FAP or, Gardner’s syndrome, so the tumor was considered to be sporadic. Immunohistochemically, it was positive for β-catenin and negative for CD117 (c- kit). The histological and immunohistochemical findings are shown in Fig. 2.

The postoperative period was uneventful. A close follow-up with laboratory tests and ultrasound was performed every 6 months and computed tomography (CT) was carried out annually for 2 years. Subsequently, all the examinations were performed once a year. Until now (4 years since the operation), the patient has had no signs of recurrence.

## Discussion and conclusions

According to their location, desmoid tumors are divided into extra-abdominal, intraabdominal, and in the abdominal wall [3]. Church (1995) classified them into four groups: tumors that resolve spontaneously (10% of cases), tumors with cycles of progression and resolution (approximately 30%), tumors that remain stable (approximately 50%) and neoplasms with rapid, aggressive progression (10%) [6]. In approximately 8% of patients, the disease can lead to death as a result of recurrence [7].

The diagnosis of intraabdominal desmoid tumors is usually delayed due to the lack of specific symptoms and often asymptomatic behavior. The clinical presentation can be very diverse and can lead to serious, sometimes fatal complications. Two main tumor characteristics are responsible for this diversity but explain the most common presentations. First, the pure mass effect of the growing tumor with compression of the surrounding structures causes symptoms such as ureteric obstruction with hydronephrosis, intestinal obstruction, and vascular or neural compression. Second, direct infiltration of the surrounding tissues can lead to ischemia, perforation, fistula formation in hollow organs, and gastrointestinal or intratumoral bleeding [3, 8–12].

Cases of aggressive fibromatosis with bleeding into the abdominal cavity are sporadic. Usually, this complication results from direct invasion of major vessels and subsequent rupture. We found only one such case reported by C. Georgiades et al. (2012), demonstrating a 1.5 cm × 0.5 cm retroperitoneal tumor strangulating the splenic artery and branches of the artery to the pancreas [13]. We could not find any case presenting with intraabdominal hemorrhage due to mesenteric or small bowel fibromatosis, as in our case. Jian Li et al. (2019) reported another unusual presentation of intestinal perforation and purulent peritonitis as an onset of tumor symptoms. Again, our case was different because there was no perforation of a hollow organ but there was a rupture of the tumor pseudo-capsule with subsequent hemoperitoneum. Clinical signs such as aortic rupture, intra-abdominal abscess, or hepatic pneumatosis have also been reported [11–14].

In cases of a suspected soft-tissue tumor, the diagnostic process has to include ultrasound and CT/MRI for determination of the size and affected structures, which is the base of adequate preoperative strategy for treatment. The differential diagnosis of desmoid tumors with intra-abdominal localization is quite wide and includes gastrointestinal carcinomas, lymphomas, stromal tumors (GIST), solitary fibrous tumors, inflammatory myofibroblastic tumors, sclerosing mesenteritis, and retroperitoneal fibrosis [15]. In cases of complicated aggressive fibromatosis, the differential diagnosis is even more difficult because more common reasons for acute surgical abdomen can usually be suspected. Furthermore, the unusual presentation of the disease, mimicking acute appendicitis or cystitis, makes our report valuable for clinical practice. In our case, the neoplasm had eroded a blood vessel. The consequent bleeding in the tumor resulted in hyperextension of the pseudo-capsule and subsequent rupture with effusion in the abdominal cavity. Additionally, the proximity of the jejunal loop affected by the tumor to the ileocecal junction led to the typical localization of the abdominal pain, which is characteristic of appendicitis. At the same time, the necrotic zone of the tumor capsule with the diffusion of hemorrhagic exudate caused a local reactive inflammation of the region and the symptoms of cystitis or spreading peritonitis in the pelvis. Unfortunately, our ultrasonographic exam did not detect tumor formation, and in our differential diagnosis, we did not consider the possibility of a tumor. The best course of action should have been to perform a CT scan, but the destructive appendicitis was a highly probable diagnosis in our case, so further imaging tests were not performed, and we chose emergency surgery.

In contrast to our case, for patients with asymptomatic, nonprogressive desmoid tumors the front-line approach is a dynamic monitoring with a type of wait-and-see policy. This strategy is applicable because of the possibility of self-limitation of the tumors, even their spontaneous regression [5, 8, 9].

When a symptomatic, resectable desmoid tumor is diagnosed, especially in cases with an intraabdominal location, the treatment is mainly surgical, and the purpose is complete tumor removal with negative microscopic margins [5, 8, 9]. Therefore, the intraoperative frozen sections are useful for evaluating the resection margins [16].

Radiotherapy can be used in advanced unresectable cases, recurrent tumors or in suspicious resection margins with R1/R2 resection, but after careful evaluation of the possible benefits and complications and only after medical treatment has been considered. Nuyttens et al. (2000) have shown a beneficial role of radiotherapy in the local control of tumors [17]. Radiotherapy is significantly more effective at a total dose > 50 Gy, but high rates of radiation-related complications are associated with doses > 56 Gy [17]. Complications of radiotherapy are established in 22.8% of cases [15]. Because of bowel toxicity, radiotherapy is rarely used for the intraabdominal localization of the disease.

Systematic treatment options include chemotherapy, antihormonal therapies, NSAIDs and tyrosine kinase inhibitors [5, 18–21]. The usage of NSAIDs is related to their limited toxicity and low costs [5]. Indomethacin and sulindac are the most common choice [22]. Antihormonal therapy, usually with tamoxifen, is also used. Chemotherapy is advisable in patients with hormonal therapy failure, aggressively growing, symptomatic or even life-threatening desmoid tumors and in cases with critical anatomic site [5]. Methotrexate and vinblastine are used mainly in the pediatric-patient population, and in adults, vinblastine has been replaced with vinorelbine because of its lower toxicity [23–25]. It has been reported that pegylated liposomal doxorubicin is a possible option [5]. A phase II study by Liu X (2018) et al. showed that a doxorubicin-based regimen with thalidomide could offer an effective and well-tolerated option for the treatment of patients with refractory AF [26]. Locoregional chemotherapy has a place in the treatment of AF, especially in cases where the disease is localized in the extremities. Targeted therapies with imatinib and sorafenib have also been investigated, but there is no randomized study showing their benefits. A recently published noncomparative, randomized, open-label, multicenter, phase 2 study (DESMOPAZ) demonstrated that pazopanib is a viable option for progressive desmoid tumors [27]. Regardless of the selected therapeutic strategy, the recurrence rate remains high – 24 – 77% [28].

In conclusion, aggressive fibromatosis remains a serious problem with the possibility of locally aggressive behavior with high rates of recurrence. Sometimes, as shown by our patient, the clinical and macroscopic recognition of aggressive fibromatosis can be immensely tricky, especially in patients presenting with symptoms of acute surgical abdomen.

## Abbreviations

AF: Aggressive fibromatosis
CT: Computed tomography
FAP: Familial adenomatous polyposis
MRI: Magnetic resonance imaging
NSAIDs: Nonsteroidal anti-inflammatory drugs
